# The efficacy of a targeted PREVENTION programme for addictive behaviour (PREVENTURE) among vulnerable ADOlescents in France - study procotol

**DOI:** 10.1186/s12889-021-10795-9

**Published:** 2021-04-23

**Authors:** morgane guillou-landreat, Elkhan Tahmazov, Schreck Benoit, Marie Grall-bronnec, Patricia Conrod, Audrey Livet, Emmanuel Nowak, Jean -Yves Le Reste

**Affiliations:** 1 EA 7479 SPURBO, University of Brest, Addictive disorder centre, CHU BREST HUGOPSY network, Rennes, France; 2 Addictive disorders Unit, CHU Brest, University of Brest, Brest, France; 3 Addiction and Psychiatry Department, CHU Nantes, Nantes, France; 4 INSERM UMR 1246, SPHERE, Methods in Patient-Centred Outcomes and Health Research, Nantes and Tours University, Nantes, France; 5 HUGOPSY network, Rennes, France; 6 CHU Sainte-Justine Research Center, 3175 Côte Ste-Catherine, Montréal, H3T 1C5 Canada; 7 Department of Psychiatry and Addiction, Faculty of Medicine, Université de Montréal, Montréal, Canada; 8 Centre d’Investigation Clinique-INSERM CIC 1412, CHRU, Brest, France; 9 EA 7479 SPURBO, University of Brest, Brest, France

**Keywords:** Alcohol, Alcohol use disorder, Targeted alcohol use prevention

## Abstract

**Background:**

Alcohol use is a major public health challenge in France, where at the age of 17 half the young people report an episode of severe alcohol intoxication in the month preceding the survey. Numerous prevention programmes have a general objective, but in adolescence individual vulnerabilities towards addictions differ significantly with personality traits. Prevention targeting personality traits enables work on risk factors for addictive behaviours, and has shown genuine efficacy.

Among existing programmes, PREVENTURE has shown an effect on the reduction in alcohol consumption by targeting four personality traits: impulsivity, sensation-seeking, negative thoughts and anxiety. This programme has been tested on samples recruited in adolescent populations in school environments, identifying adolescents at risk, but it has not been tested on a more targeted recruitment of adolescents seen in consultation.

**Methods:**

The main hypothesis of this study is that the targeted prevention programme PREVENTURE will have an impact on the prevalence of binge-drinking episodes. The secondary hypotheses explore other factors such as associated substance use, anxiety and depression, as well as the acceptability of the programme. This article presents the study protocol of “PREVADO” study.

We intend to assess the impact of the targeted intervention programme PREVENTURE on the prevalence of binge-drinking episodes among adolescents aged 14 to 17 years consulting in one of the participating centres or referred by a school doctor. The study will be prospective, randomised, controlled and open-label, and will comprise an intervention group and a control group.

The adolescents will then be followed and assessed 1, 3, 6 and 12 months after the intervention.

The study needs to include 700 subjects in order to reach 340 adolescents randomised, 170 in each group. It will concern 33 centres.

**Discussion:**

This project could favour the targeting of addictive behaviours among vulnerable adolescents, and its application on a larger scale could be envisaged.

**Trial registration:**

The Trial registration number is NCT04599270, and it was registered on the ClinicalTrials.gov public website.

## Background

Alcohol use is considered as a leading risk factor in the global burden of disease and causes substantial damage to health [1–3]. Four per cent of the global burden of disease is attributable to alcohol [4]. Excessive alcohol consumption results in 3.3 million deaths each year, 5.9% of all deaths around the world [5]. Alcohol consumption tends to emerge during adolescence, and is especially risky for adolescents given its impact on the developing brain [1]. Moreover, from the very first episodes of alcohol consumption, intensive binge drinking can cause severe damage in the short and medium to long term [2, 3], such as alcohol-induced coma, involvement in interpersonal violence as a victim and/or as a perpetrator, non-consensual sexual acts, road accidents, etc. [4, 5]. Evidence suggests that an early onset of alcohol use increases the risk of alcohol use disorders in later life and comorbid mental health problems [6].

In Europe, in 2019, 79% of students reported having consumed alcohol at least once during their lifetime and 13% of the students in the ESPAD study reported having been intoxicated in the last 30 days prior to survey [7]. In France more particularly, nearly 80% of those aged 14 had already experimented alcohol in their lifetime [8], and half of the adolescents aged 17 reported a binge drinking episode in the last month (over 5 standard drinks on the same occasion) [9].

Social and cultural factors influence consumption and lead to a social valuation of alcohol consumption and binge drinking [9, 10]. There are also individual vulnerability factors, particularly marked in adolescence in the transition from experimentation to regular use or even alcohol use disorders (AUD) [11]. Despite the extent of the damage, there is currently little reliable data on efficacious primary prevention strategies for dealing with addictive behaviour [12]. Many prevention programmes target age groups in schools, to delay or reduce the use of psychoactive substances or the harm associated with their use. However a meta-analysis on the impact of school-based prevention programmes concluded that most interventions were associated with little or no impact in terms of reducing substance misuse among adolescents [13].

During adolescence, individual vulnerability with regard to the risk of addictive disorders differs significantly according to personality traits [14]. Targeted prevention on the basis of personality entails work on the risk factors of addictive behaviour (tobacco, alcohol, cannabis, etc.) and has proved to be highly efficacious in terms of reducing consumption, damage and alcohol acceptability among adolescents [15].

Among existing programmes, the *Preventure* program has been evaluated in 5 trials with high-risk adolescents identified in school settings accross different countries (Canada, Europe, Australia) [10–14]. *Preventure* is a preventative program that utilizes Cognitive behavioural therapy (CBT) techniques and motivational interviewing (MI) strategies. This program is selectively administered in schools to adolescents identified as at risk for addictive disorders by targeting four personality risk factors: negative thinking, anxiety sensitivity, impulsivity and sensation-seeking [15]. Preventure was originally designed to target substance use prevention and findings across the literature have demonstrated clear and robust effects on the reduction of alcohol consumption [8, 16–19]. This programme has been studied in different adolescent samples recruited from the community (i.e., schools), identifying at-risk adolescents.

Nevertheless, *Preventure* has never been tested among adolescents who are neither seeking treatment nor have been identified as at risk by school nurses or medical doctors. A recent review highlighted the need to make this program more accessible by targeting adolescents pre-identified as vulnerable and by studying the impact of the programme on such populations [15]. This strategy could enable adolescents who have left school and adolescents with higher vulnerabilities to have access to the program.

The current randomized controlled trial was designed to evaluate the impact of the *Preventure* program on adolescents (i.e., age between 14 and 17), in comparison to usual follow-up care for adolescents (i.e., identified in primary care, or in adolescent care or through school nurses or doctors).

The main hypotheses of this study are that 1. the *Preventure* program will potentially have an impact on 1/ the prevalence of binge drinking episodes 2/ the number of days of alcohol consumption, 3/ associated substance uses: tobacco/ cannabis, 4/ anxiety and depression, 5/ alcohol-related harm, and 6/ quality of life. Moreover, it is also hypothesized that the Preventure program will be considered as an acceptable intervention among French adolescents.

This project could promote the targeted prevention of addictive behaviours among vulnerable adolescents. Thus, the use of the Preventure program could be considered on a larger scale and developed in primary care and schools or adolescent care facilities in France.

This article presents the study protocol; it follows the Standard Protocol items: recommendations of the 2013 Interventional Trial (SPIRIT) guidelines [16].

## Methods/design

### Aim of the study

The main objective of the current study is to evaluate the impact of the *Preventure* program on the prevalence of binge-drinking episodes (i.e, defined as more than 5 standard units of alcohol on a single occasion) among adolescents aged between 14 to 17 and at risk of developing problematic alcohol use, in comparison with usual follow-up care. The secondary objectives are to assess the impact at different times of follow up (3, 6 and 12 months) on 1/ the number of days with alcohol consumption 2/ associated substance uses: tobacco/cannabis, 3/ anxiety and depression, 4/ alcohol-related harm, 5/ quality of life at each stage, 6/ the acceptability of the intervention among French adolescents.

### Study setting

This study will take place in the western region of France, in primary care settings, in specialized adolescent care settings and in schools (school nurses and doctors). Information and poster campaigns will be disseminated within the networks, to the schools concerned and to the general public. This dissemination will enable the public concerned to be broadly informed.

### Participants and materials

All adolescents aged 14 to 17 years seeking treatment, for any reason, in the networks involved or identified by the school medical staff and meeting the inclusion criteria will be offered the opportunity to participate in the study.

### Inclusion criteria

Adolescents aged 14 to 17 years, seeking treatment in one of the participating centres in primary care, or adolescent care facilities (general or addictive disorder consultations for adolescents) or identified by a school doctor or nurse, who have already experienced alcohol (at least 1 standard unit of alcohol lifetime), will be included in the study, after written and informed consent from the parents and the adolescents. They are required to have Internet access, access to a smartphone, tablet or computer.

### Non-inclusion criteria

The non-inclusion criteria will be the following: adolescents expressing refusal to participate in the study, or presenting moderate-to-severe substance use disorders (other than tobacco) or current non-stabilized psychiatric disorders, or current treatment for severe substance use disorders and/or severe psychiatric disorders (psychotic disorders, autistic disorders), or inadequate understanding of the French language. A lack of internet access, access to a smartphone, tablet or computer will also be a non-inclusion criterion.

### Exclusion criteria

Adolescents who are not at risk of developing problematic substance use according to the negative thinking, sensitivity to anxiety, impulsivity and sensation-seeking dimensions of the SURPS (Substance Use Risk Profile Scale) will be excluded from the study (cut-offs: ≥14, ≥17, ≥15, ≥13 respectively) [8].

### Consent

The investigator will provide complete verbal and written information to the patient and parents, checking the inclusion and non-inclusion criteria. Once eligible adolescents and their parents who have given their verbal consent, they will be referred to the study psychologist who will obtain the written consent of the parents and the adolescent. Subjects will be able to withdraw their consent and request to be removed from the study at any time for any reason. The investigator may temporarily or permanently discontinue a subject’s participation in the study for any reason that would be in the best interests of the subject, particularly in the case of serious adverse events. If a subject is lost to follow-up, the investigator will make every effort to re-establish contact with the person. In the case of a withdrawal of consent, the data collected will not be used in accordance with the patient’s wishes.

During their participation, patients will not be able to participate in research on addictive behaviours, and there are no restrictions on treatment.

An assessment of personality characteristics that represent a risk for the development of problematic substance use using the SURPS questionnaire will be carried out. Only adolescents with risk-related personality traits, according to at least one of the dimensions: negative thinking, sensitivity to anxiety, impulsivity and sensation-seeking on the SURPS, will be randomized. The others will be excluded from the randomized trial. Adolescents excluded prior to randomization will have a meeting with a psychologist to discuss the risks associated with addictive disorders and they will be given information about risk prevention.

### Intervention

The *Preventure* program consists of two 90-min sessions with delivered to small groups of students (i.e., approximately 4–6 students per group), led by a trained facilitator. In 2020, this program was adapted for online delivery via videoconferencing platforms and can now be accessed by adolescents on smartphones, tablets or computers. This online program enables wider access to the intervention for adolescents, whether they live in rural, semi-urban or urban areas .

Supported by real-life scenarios, and based on CBT and MI techniques, the sessions will incorporate:

1) Psychoeducation about the targeted personality trait (i.e., impulsivity, sensation seeking, anxiety sensitivity, hopelessness/negative thinking) and associated maladaptive coping behaviours.

2) The development of an individualized model of the physiological, cognitive and behavioural components of typical responses.

3) Motivational enhancement, goal setting and application of cognitive-behavioural skills to tackle problematic reactions and behaviour.

Each adolescent will receive the intervention corresponding to their predominant personality trait.

### Study design

This is a prospective, randomized, controlled, stratified, multicentre, open-label study aiming to evaluate the superiority of the *Preventure* program on the reduction of binge drinking episodes, over usual routine follow-up care forming the control group (individual follow-up consultations in primary care/or in adolescent care facilities/ adolescent addictive disorder consultations). It concerns a population of adolescents aged 14–17 having experimented with alcohol consumption.

**Fig. 1:**
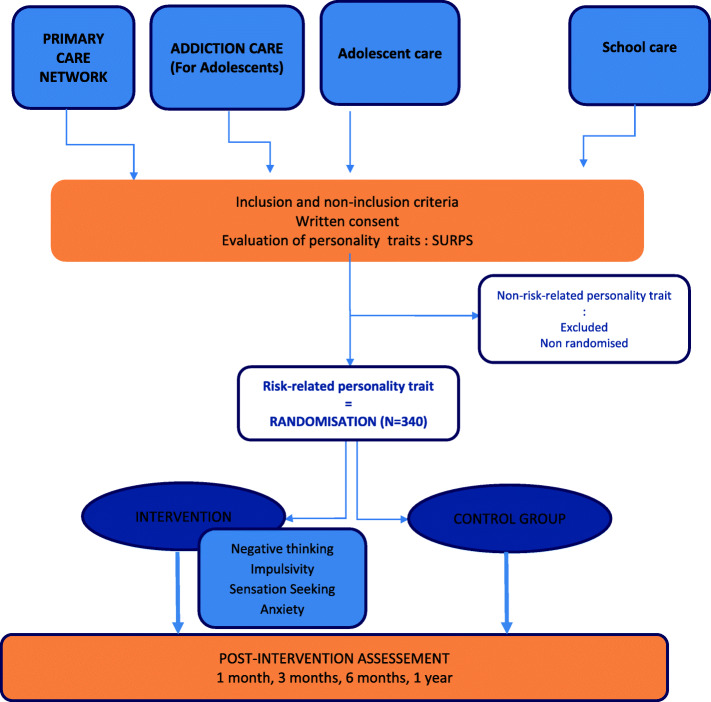
Study design

The study design is presented in Fig. 1.

### Randomisation

Adolescents will be randomised [1], and the randomisation will be stratified according to the predominant type of risk-prone personality trait (4 types) presented by the adolescents and according to recruitment conditions (school care vs. primary or specialised adolescent care). The control group will receive the usual routine follow-up (individual consultations in primary care / or in adolescent care facilities/ adolescent addictive disorder consultations) and assessments.; The intervention group will receive the usual follow-up, assessments plus the Preventure program tailored to one personality type among the 4 risk-related personality traits.

### Follow-up assessments

Adolescents will then be followed up and evaluated at 1, 3, 6 and 12 months after the intervention by a psychologist. Assessments will be offered to adolescents either by phone or during an interview lasting approximately 1 h, depending on their preference.

### SURPS: substance use risk profile scale

The Substance Use Risk Profile Scale (SURPS) is a screening instrument for personality traits identified as related to the risk of developing of addictive disorders. The SURPS consists of 23 items assessing four dimensions: Impulsivity, sensation-seeking, negative thinking and anxiety. The SURPS was created by Woicik et al. [17] and includes 23 items, to which young people are asked to respond using a 4-point Likert scale, ranging from strongly agree to strongly disagree: negative thinking (7 items), sensitivity to anxiety (5 items), impulsivity (5 items) and sensation-seeking (6 items).

The four different personality dimensions measured are associated with a high risk of developing addictive disorders. It was validated in French on a population of students from Quebec. The psychometric properties of the French version were satisfactory and comparable to previous studies and to the original version. The thresholds defined as risk-related were calculated according to means and standard deviations, and for the French version are: Negative thoughts = 15; Anxiety = 13; Impulsivity = 14; Sensation seeking = 17 [8].

### **Alcohol- timeline follow-back (TLFB)** [18]

The Alcohol-TLFB is a method of assessing alcohol consumption that provides an estimate of the daily amount of alcohol consumed in standard units (equivalent to 10 g. alcohol per drink). It has been evaluated among clinical and non-clinical populations and among adults and adolescents. It is presented in the form of a calendar.

Respondents report their daily consumption of alcohol over a specified period of the last 30 days. Reminders can be used when filling in the form (dates, events, etc.). This tool has good psychometric qualities and is recommended when precise information on the quantities of alcohol consumed or the number of days of consumption is required. It enables the number of days of alcohol consumption in the month and the number of days with alcohol consumption in excess of 5 standard units per day to be identified.

### **ADOSPA** [19]

The ADOSPA test is a French adaptation of the CRAFFT. It is a simple test for early detection of risky and harmful alcohol or drug use among young people. It consists of 6 questions. A score above 3 indicates high risk.

### **PedsQL4: Paediatric quality of life inventory version 4.0** [20]

The PedsQL 4.0 is a tool evaluating the quality of life of children, adolescents, or young adults (2 to 25 years old) in good health or with health problems. The 23 questions cover 4 different functioning areas: physical (8 items), emotional (5 items), social (5 items) and academic (5 items).

It is a questionnaire that can be self-administered, with the addition of a questionnaire dedicated to family and friends. The questionnaire intended for parents and relatives enables the parents to give an account of their perceptions regarding the quality of life related to their child’s state of health. It takes the child’s perceptions and those of his parents or relatives into account.

### **DSM 5: diagnostic and statistical manual of mental disorder** [21]

The diagnosis of substance use disorder is based on well-defined criteria set out in the Diagnostic and Statistical manual of Mental disorders (DSM), the fifth edition of which dates from 2013. The severity of SUD is classified as: mild (2 to 3 criteria) / moderate (4 to 5 criteria)/ severe (6 or more criteria).

### **AUDIT: alcohol use disorders identification test** Error! Bookmark not defined.

This is a simple 10-question test developed by the World Health Organisation to determine whether a person is at risk for alcohol addiction. A score between 7 and 12 for men and between 6 and 12 for women indicates risky or problematic drinking. A score above 12 indicates probable alcohol use disorder.

### **HONC: hooked on nicotine checklist** [22]

Di Franza’s Honc checklist (Hooked on Nicotine Checklist) is a 10-item test that highlights the loss of control over tobacco consumption. This tool is particularly suitable for young smokers. The scoring ranges from 1 to 10 with yes-no answers. A positive response to a single item is sufficient to indicate a loss of autonomy towards tobacco consumption. The higher the total number of positive responses, the more the test result suggests a loss of autonomy and therefore dependence. A score of 7 and above indicates a high level of dependence.

### **CAST: Cannabis abuse screening test** [23]

The CAST is a 6-item scale for identifying problematic cannabis use. Each of the items describes behaviours or problems encountered in the context of cannabis use. A score of 2 and above means a high risk of cannabis use disorder.

### **ADRS: adolescent depression rating scale** [24]

The ADRS test assesses depression among adolescents in 10 items. The items measure insomnia, anxiety, sadness and fatigability. The ADRS score enables the identification of a low risk of depression with a threshold > 4, moderate risk for a value between 4 and 8 or significant risk for a score > 8.

### Judgment criteria

The main criterion is the occurrence of at least one binge drinking episode (at least 5 Standard (US) Units of alcohol, equivalent to 50 g of pure alcohol/unit at one time) in the last month (evaluated at 6 months after intervention) measured with the Alcohol -Timeline Followback A-TLFB) proposed by (Sobell et al. 1998).

The secondary criteria are:

- Criteria for evaluating alcohol, tobacco or cannabis consumption: experimentation / use within the month / regular (more than 10 times a month), measured with the A-TLFB.

- Evaluation of alcohol, tobacco or cannabis consumption profiles: AUDIT, HONC, CAST and ADOSPA tests - Assessment of anxiety and depression: ADRS.

- Alcohol-related harm: collection of negative events linked to alcohol consumption in the past month, by interview.

- DSM-5 criteria for substance use disorders: alcohol / cannabis.

- Quality-of-life assessment: PedsQL V4.

- Assessment of the acceptability by adolescents: participation and retention rates in the study.

### Sample size

On the basis of the results observed in the publication by Lammers et al. [13], 50% of adolescents who have had at least one binge-drinking episode in the last month are expected in the control arm while 35% are expected in the intervention group. With an alpha risk of 0.05 and a potency of 80%, the sample size should be 170 patients/group, or 340 patients in all. As the expected proportion of “at-risk” subjects (who will be randomised) is of the order of 1 in 2 among the adolescents included, an initial number of 700 adolescents is planned to be included (including a proportion of subjects not evaluated for the main criterion).

Thirty-three centres will be included, distributed across 8 hospital structures and the general medicine network, corresponding to less than one patient/month per investigator.

The duration of the inclusion period will be 36 months, the duration of participation for each patient will be 15 months, and the total duration of the study will be 51 months.

### Statistical analysis

The prevalence rates of adolescents who have experienced at least one binge drinking episode (more than 5 standard units) in the last month (measured at 6 months after intervention -main criterion) will be compared using logistic regression with adjustment for stratification factors between the two groups (intervention and control). As an exploratory measure, the interaction between the intervention and the type of risk profile will be tested, looking for possible heterogeneity of effect of the intervention according to the type of profile. In the case of a significant interaction, results by type of profile can be provided on an exploratory basis.

The binary secondary judgement criteria (pass/fail) will be analysed in the same way as the main criterion.

Quantitative judgement criteria will be analysed using a linear model with adjustment for stratification factors. The interaction term between intervention and profile type can be tested in the same way as for the main criterion.

Quantitative measurements repeated during monitoring will be modelled using a mixed linear model to take intra-subject correlation into account.

### Harms

The study may be stopped prematurely in the event of unexpected, serious adverse events requiring a review of the strategy. Unexpected or serious events will be collected by investigators.

### Data management and confidentiality

Data entry will be carried out on an electronic medium via a web browser. Data analysis will be carried out by a biostatistician from the Data Management Unit (CHRU BREST) under the responsibility of Emmanuel NOWAK.

Persons with direct access will take all necessary precautions to ensure the confidentiality of information relating to the products, the tests, the persons who take part in them and in particular with regard to their identity and the results obtained. These persons, in the same way as the investigators themselves, are subject to professional secrecy (according to the conditions defined by articles 226–13 and 226–14 of the French penal code).

During the research or at its end, the data collected on the persons who are subject to such secrecy and transmitted to the sponsor by the investigators (or any other specialized contributors) will be made anonymous. Under no circumstances should the names or addresses of the persons concerned be made clear. Only the first letter of the subject’s surname and the first letter of the subject’s first name will be recorded, accompanied by a coded number specific to the study indicating the order of inclusion of the subjects. The sponsor will ensure that each person involved in the research has given written consent for access to individual data concerning him or her that is strictly necessary for the quality control of the research.

### Authorship guidelines

The scientific communications and reports corresponding to this study will be carried out under the responsibility of the investigator coordinating the study with the agreement of the scientific committee. The co-authors of the report and publications will be the investigators and clinicians involved, in proportion to their contribution to the study, as well as the biostatistician and associated researchers. The publication rules will follow international recommendations (N Engl J Med, 1997; 336: 309–315). The study will be registered on a freely accessible website (Clinical trial) before the inclusion of the first patient in the study.

### Dissemination policy

The results will be communicated to the general public, through local and regional media and social networks, but also in a more targeted manner to the participants through a specific communication disseminated to the participating centers. In addition, open access scientific journals will be preferred for the publication of results.

## Discussion

Alcohol use causes substantial harm to young people and has an impact on the wider general public [25]. The development of an efficacious prevention programme for addictive disorders is paramount to reduce the burden and the cost of alcohol use. In 2018, the French MILDECA (Inter-ministerial mission for the fight against drugs and addictive behaviour) published a report “Young addicts and prevention” in 2018 highlighting the importance of developing efficacious, validated and readily-assessable prevention programmes in France. They underlined the need to transpose programmes recognized as efficacious in other countries to a French context by favouring multidimensional approaches [4].. This project is in line with these current priorities, as it directly concerns the targeted prevention of addictive behaviours among adolescents at risk. It involves adolescent primary care, specialised adolescent care, and the school environment (school nurses and doctors), i.e. all places potentially frequented by the age group concerned (14 to 17 years)..

There is currently no intervention or programme that is systematically offered to adolescents with risk-prone personalities in this particular age group in France. Individual benefits will be assessed by criteria concerning psychoactive substance use and impact on quality of life.

The expected individual and collective benefits are significant, with the development of a validated programme having already shown a very positive individual and collective impact on adolescent populations [11, 13]. Excessive alcohol consumption and alcohol use disorders are highly prevalent problems and generate severe damage [26, 27]. There is currently no validated, structured prevention programme on offer to the various players who work with adolescents. If this study can demonstrate the efficacy of this programme on this population, with a multicentre recruitment involving primary care, adolescent care and school nurses and doctors, the implementation of this type of programme could be envisaged on a larger scale and could be systematized. We could demonstrate the interest and feasibility of multi-level inclusion involving any kind of facility catering for adolescents.

### Strengths and limitations

The main strength of this study is the methodology: a prospective, randomized, controlled design, which will provide a high level of evidence on the efficacy of the programme. In addition, the *Preventure* programme has mostly been deployed in schools, but deployment of evidence-based programmes for problematic alcohol use in a school setting is limited, and there are several barriers to implementation, such as cost, geographical restrictions, and access to training [28]. The protocol for this study is original because *Preventure* will target adolescents referred by a care network (primary care, adolescent care, school nurses and doctors). Indeed, targeting vulnerable populations for this programme proves necessary, as this review highlights [15].

The current study is not without limitations. As with most research exploring adolescent substance use, our outcome measures are reliant on student self-report. Their responses may be subject to bias. However, studies have demonstrated self-report to be a valid method for assessing adolescent substance use symptoms [29], and students are informed that their responses are confidential. There may be participant attrition, given the longitudinal follow up of the study design.

### Implications

Alcohol-related disorders are complex: vulnerability factors are a combination of individual factors (gender, psychiatric disorders, family history of addictive disorders, personality traits, etc.); environmental factors (social integration, social entertainment, work and family status, etc.) and alcohol-related factors (type of alcohol, social status, harm, etc.). The aim of this programme is to work on individual factors: personality traits. It will seek to help adolescents to know how they function in order to be able to tackle their alcohol problems. In the long term, if the inclusion of at-risk adolescents via the networks of primary care consultations, adolescent care and school medical staff proves feasible and if the program shows its efficacy, it will be interesting to continue the work by way of a comparative analysis between the PreVenture programme and the PreVenture programme complemented by the active involvement of primary care physicians to sustain the benefits of the programme.

## Data Availability

No complementary data available.

## Abbreviations

API: Alcoolisation Ponctuelle Importante
A-TLFB: Alcohol –Timeline Followback
SURPS: Substance Use Risk Profile Scale
CBT: Cognitive Behaviour Therapy
MI: Motivational Interviewing
